# Stability and Free Radical Production for CO_2_ and H_2_ in Air Nanobubbles in Ethanol Aqueous Solution

**DOI:** 10.3390/nano12020237

**Published:** 2022-01-12

**Authors:** Zhenyao Han, Hiromi Kurokawa, Hirofumi Matsui, Chunlin He, Kaituo Wang, Yuezou Wei, Gjergj Dodbiba, Akira Otsuki, Toyohisa Fujita

**Affiliations:** 1 School of Chemistry and Chemical Engineering, College of Resources, Environment and Materials, Guangxi University, Nanning 530004, China; 1622303001@st.gxu.edu.cn (Z.H.); helink1900@126.com (C.H.); wangkaituo@gxu.edu.cn (K.W.); 2 Algae Biomass Energy System R&D Center (ABES), University of Tsukuba, Tsukuba 305-8572, Japan; h.kurokawa@mobiol.tech; 3 Faculty of Medicine, University of Tsukuba, Tsukuba 305-8575, Japan; hmatsui@md.tsukuba.ac.jp; 4 School of Nuclear Science and Technology, University of South China, Hengyang City 421001, China; yzwei@usc.edu.cn; 5 Graduate School of Engineering, The University of Tokyo, Bunkyo 113-8656, Japan; dodbiba@g.ecc.u-tokyo.ac.jp; 6 Ecole Nationale Supérieure de Géologie, Geo Ressources UMR 7359 CNRS, University of Lorraine, 2 Rue du Doyen Marcel Roubault, BP 10162, 54505 Vandoeuvre-lès-Nancy, France; akira.otsuki@univ-lorraine.fr; 7 Waste Science & Technology, Luleå University of Technology, SE 971 87 Luleå, Sweden

**Keywords:** nanobubble stability, free radical, carbon dioxide, hydrogen, ethanol aqueous solution, extended DLVO theory

## Abstract

In this study, 8% hydrogen (H_2_) in argon (Ar) and carbon dioxide (CO_2_) gas nanobubbles was produced at 10, 30, and 50 vol.% of ethanol aqueous solution by the high-speed agitation method with gas. They became stable for a long period (for instance, 20 days), having a high negative zeta potential (−40 to −50 mV) at alkaline near pH 9, especially for 10 vol.% of ethanol aqueous solution. The extended Derjaguin, Landau, Verwey, and Overbeek (DLVO) theory was used to evaluate the nanobubble stability. When the nanobubble in ethanol alkaline aqueous solution changed to an acidic pH of around 5, the zeta potential of nanobubbles was almost zero and the decrease in the number of nanobubbles was identified by the particle trajectory method (Nano site). The collapsed nanobubbles at zero charge were detected thanks to the presence of few free radicals using G-CYPMPO spin trap reagent in electron spin resonance (ESR) spectroscopy. The free radicals produced were superoxide anions at collapsed 8%H_2_ in Ar nanobubbles and hydroxyl radicals at collapsed CO_2_ nanobubbles. On the other hand, the collapse of mixed CO_2_ and H_2_ in Ar nanobubble showed no free radicals. The possible presence of long-term stable nanobubbles and the absence of free radicals for mixed H_2_ and CO_2_ nanobubble would be useful to understand the beverage quality.

## 1. Introduction

There have been many reports on bulk nanobubbles or nanoparticles in ethanol aqueous solution. In this research, the object of nanobubbles in ethanol solution was hydrogen in argon and carbon dioxide gas nanobubbles. However, there are some reports on the exsistence of imputities in bulk ethanol nanobubbles. Therefore, they were first introduced and then the other reports on the stability of nanobubbles in ethanol were discussed.

The structure and properties of ethanol aqueous solutions were reported in the literature [1,2,3,4]. For nanobubble production in ethanol aqueous solution, some works have reported existing impurities. Rak and Sedlák, 2020 showed the existence of hydrophobic impurities in mixtures [5]. Alheshibri and Craig, 2019 reported that the ethanol–water mixture was found to produce only positively buoyant ethanol and water particles, with a mean density of 0.91 ± 0.01 g/cm^3^. Resonant mass measurements suggested that suspended ethanol and water particles produced by mixing water and organic solvent had a higher density than a bubble, but smaller than ambient water [6]. The bulk nanobubbles adsorb impurities and concentrate them on their interfaces with bubbles [7]. The nucleation dynamics during the water–ethanol–water exchange was reported as follows: within 4 min after the exchange, the bubbles nucleated and formed a stable population, and the tracer particles concentrated near the nanobubbles as a result of Brownian motion [8]. The low concentration nanobubble at 10^8^/mL was produced from saturated CO_2_ at 1 atm. The solutions scattered light for a long period (days) after mixing, and scattering objects originated from water-insoluble impurities in 20 vol.% ethanol [9]. A small amount of hydrophobic material was dissolved in the commercial ethanol, and the hydrophobic organic nanodroplets in the alcohol–water substitution method were often misunderstood to be nanobubbles [10].

Jadiv and Barigou, 2020 reported that, in a water and ethanol mixture, the nano- materials generated by hydrodynamic cavitation and ultrasound cavitation were not ethanol droplets, and the amount of dissolved gas directly affected the number and stability of nanobubbles generated [11]. The maximum value in the adsorption isotherm of ethanol is expected to form a monolayer of ethanol [12]. The direct arrangement of ethanol molecules can be induced by these interfaces owing to the amphiphilic properties of ethanol molecules and the stabilization of bulk nanobubbles [13,14]. Tan et al., 2021 reported the surface and bulk nanobubbles, and they were thermodynamically stable [15]. Chen et al., 2021 reported that, in the air bubble composition in the ethanol aqueous solution, ethanol molecules were preferentially oriented at the ethanal–water interface, and their orientation apparently did not vary with alcohol concentration, while water molecules were arranged to maximize the hydrogen bonding between the oriented alcohol and the adjacent water molecules [16]. When the ethanol was added to water, the ethanol dissolved rapidly, and the dissolved air in ethanol diffused slowly. Therefore, the local supersaturation of air in the solution formed tiny bubbles [13,17]. Chen et al., 2021 described that the observed “colloidal particles” in their ethanol–water solution prepared by a controllable bulk mixing method were nanobubbles rather than contaminant particles [16]. There are many models for nanobubbles; however, the charge stabilization model can provide reasonable and consistent explanations for the three properties (i.e., 100 to 1000 nm radii, the strict maximum limit for the bubble size, and the increase in implied radius with the ionic concentration) claimed by the dynamic light scattering (DLS) experiments [18]. Although ethanol is important for the formation of nanobubbles, the addition of excess ethanol higher than 20 vol.% may cause the nanobubbles to disappear. This result is consistent with the change in the long-range hydrophobic force with ethanol contents [19]. The long-range attraction between hydrophobic surfaces is due to bridging of sub-micron bubbles. Sun et al., 2021 suggested that it is impossible to judge whether the nanoparticles are nanobubbles according to a single condition. Thus, researchers should combine multiple physical properties (i.e., volume change, density, zeta potential, and refractive index of nanoparticles) to verify the presence of nanobubbles. In addition, a better understanding of interface theory can help researchers to distinguish them. It is suggested that the growth or contraction of bulk nanobubbles can change the surface tension, which can be altered by the interfacial adsorption of pollutants. Therefore, the most important thing is to avoid the interference of pollutants [20].

Hydroxyl radical production is well-known for the Fenton reaction [21], while nanobubbles in a solution that does not contain ferrous ions are discussed. First, Takahashi et al., 2007 reported on the hydroxyl radical (∙OH) generation from collapsing microbubbles [22], and his group then reported that ∙OH was produced in both cases of air (oxygen microbubbles and nitrogen microbubbles) in the acidic condition at pH 2 and 3 [23]. Moreover, the microbubbles on ozonized water indicated the production of ∙OH by the collapse of the microbubbles [24]. In 2021, they reported that ∙OH was generated from the collapsing microbubbles under strongly acidic conditions without any dynamic stimulus such as ultrasound or a large pressure difference [25]. Liu et al., 2016 reported that ·OH and superoxide ion (·O_2_^−^) can be produced in an aqueous solution with bulk nanobubbles, and investigated the germination processes for plant seeds [26]. The ·OH could be generated directly from bursting nanobubbles or simple hypoxic sediment/water oxygenation [27].

On the other hand, the free radical could not be generated by the self-collapse of air micro/nano bubbles in pure water produced by fiber membrane filter, and the ·OH peak was observed with a weak supersonic wave [28]. The results of the numerical simulations suggested that no ·OH was produced from a dissolving nanobubble. It was suggested that the signals reported experimentally did not originate in ∙OH, but rather in H_2_O_2_ produced during hydrodynamic cavitation in the production of bulk nanobubbles [26]. The radical production by ultrasonic wave irradiation becomes more important to produce the radicals, especially ∙OH in water, by comparing no irradiation and irradiation [29]. Fujita et al., 2021 reported the ∙OH scavenging and the ·O_2_ diminishing by mixing the CO_2_ nanobubbles after hydrogen nanobubble blowing in water and alcohol aqueous solution [30]. As described above, the ethanol and water solutions containing nanobubbles have various characteristics and there is a possibility to produce the free radicals. Soda drinks with carbon dioxide need long stability and the prevention of free radical production in consideration of our health.

In this study, the characteristics of hydrogen in argon and carbon dioxide nanobubbles in ethanol aqueous solution were investigated from the point of view of stability for 20 days of nanobubbles according to the surface charge on bubbles and radical production by controlling the bubble surface charge to near zero by changing pH and with the application of small ultrasonic wave and ultraviolet irradiation. When the absolute value of zeta potential of nanobubbles is low, there is a possibility of the collapse of nanobubbles by Brownian motion and the production of the free radical. The presence or absence of the free radical is useful to know the beverage quality, among others.

## 2. Materials and Methods

### 2.1. Materials

Deionized (DI) water with a resistivity of 18.2 MΩ·cm prepared by the Classic Water Purification System from Hitech instruments CO., Ltd. (Shanghai, China) was used. Ethanol with a purity higher than 99.7% produced by Guangdong Guanghua Sci-Tech Co., Ltd., Guangzhou, Guangdong, China was used. The ethanol percentages of ethanol aqueous solution mixtures were 0, 10, 30, and 50 vol%. The gases of 8% H_2_ in Ar and CO_2_ were supplied from a tank produced by Guangdong Huate Gas Co., Ltd., Guangzhou, Guangdong, China. The pH adjusters of ethanol aqueous solution were sodium hydroxide (NaOH) aqueous solution (Guangdong Guanghua Sci-Tech Co., Ltd., Guangzhou, Guangdong, China) and hydrochloric acid (HCl) aqueous solution (Chengdu Chron Chemicals Co., Ltd., Qionglai, China). The solubility of H_2_ gas [31] and CO_2_ gas [32] in ethanol was about 100 times in H_2_ and 10 times in CO_2_ larger than those gases in water.

### 2.2. Methods

#### 2.2.1. Nanobubble Preparation and Measurements

**Figure 1:**
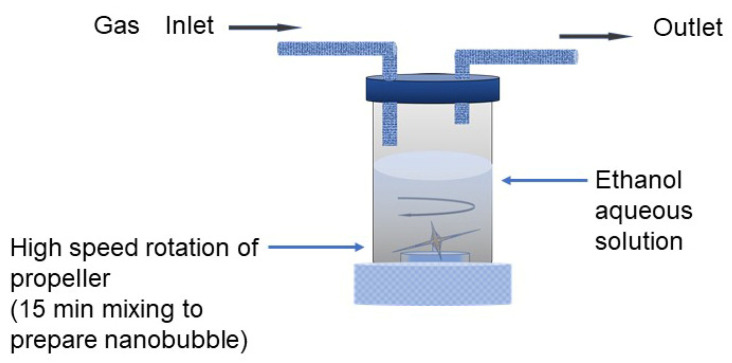
Preparation method of nanobubbles.

The nanobubbles were prepared using mechanical high-speed cavitation equipment (self-made equipment), as shown in Figure 1. The gases of 8% in Ar and CO_2_ were fed from the gas tank through the gas inlet, and the propeller mixing speed was 20,000 rpm using a 7.2 cm diameter of the blade. The gas mixture nanobubbles were prepared by initially blowing 8% H_2_ in Ar gas and then CO_2_ gas.

There are several bulk nanobubble preparation methods, such as the utilization of high-speed cavitation, pressure difference with circulation, ultrasonic wave, and passing ultrafine pores [33,34]. In this study, the equipment shown in Figure 1 was utilized to produce a large amount of nanobubbles in liquid in a fast manner. There are some methods to measure the nanobubble size; for example, dynamic light scattering (DLS), particle trajectory, resonant mass, and laser diffraction methods [34]. In particular, it was convenient to measure the particle/bubble size distribution by the DLS and particle trajectory methods discussed in previous studies of this group [35]. In this study, the nanobubble size distribution was measured by the DLS system (NanoBook Omni, Brookhaven Instruments, Holtsville, NY, USA). The nanobubble number density was obtained through outsourcing from NanoSight, NS300, Malvern (Worcestershire, UK). The zeta potential values were measured through the micro-electrophoresis method by the phase analysis light scattering method (NanoBrook Omni, Brookhaven Instruments, Holstville, NY, USA.).

#### 2.2.2. Radical Preparation and Measurement by ESR

The solution pH was adjusted to pH 9 for the three kinds of nanobubbles (i.e., 8% H_2_ in Ar, CO_2_, and a mixture of CO_2_ after 8% H_2_ in Ar) in ethanol aqueous solutions, and they were kept for 20 days. After 20 days, the pH of ethanol aqueous solutions was decreased to pH 5 by adding HCl aqueous solution, and the solutions were set in the ultrasonic wave vessel (SIBATA SCIENTIFIC TECHNOLOGY, Tokyo, Japan, SU, 40 kHz, 500 W) for 30 s; then, rapidly, the radicals were measured. The produced radicals were measured by the following procedure. A spin-trapping reagent, sc-5-(5,5-dimethyl-2-oxo-1,3,2-dioxapho-sphinan-2-yl)-5 methyl-1-pyrroline N-oxide (G-CYPMPO)24, was used by adding its solution. G-CYPMPO could spin-trap ·OH in UV (4 W, OHM ELECTRIC INC., Tokyo, Japan) illuminated condition and ·O_2_^−^ [36,37,38]. A JEOL JES-TE25X ESR spectrometer (Tokyo, Japan) was used to obtain ESR spectra of free radicals of ·OH and ·O_2_^−^. The measured peaks produced by nanobubble collapse were compared with eight kinds of peak positions of standard ·OH and ·O_2_^−^.

## 3. Results and Discussion

### 3.1. Determination of Diameter of Nanobubbles

**Figure 2:**
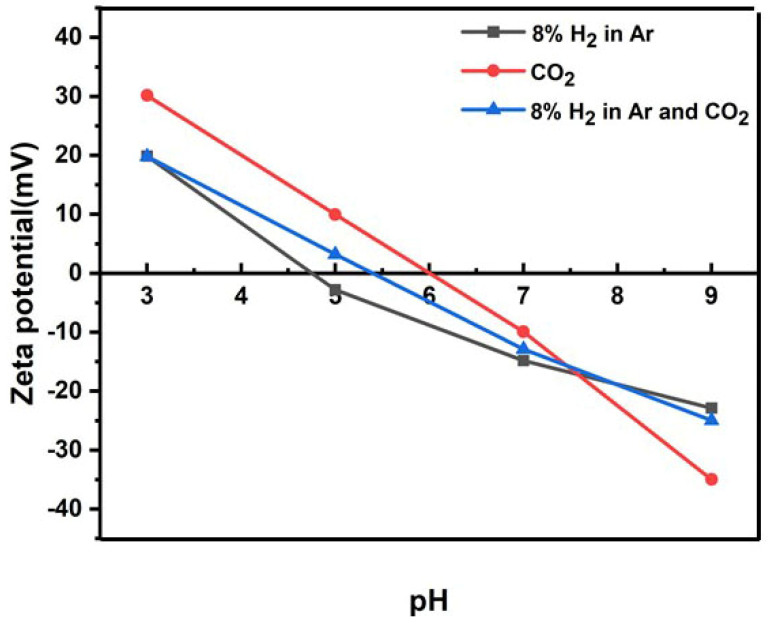
Zeta potential as a function of pH for 8% H_2_ in Ar, CO_2_, and a mixture of 8% H_2_ in Ar and CO_2_ gas nanobubble in water [35].

The stability of nanobubbles in water was reported in the literature [35], and there are reports on the stabilization of bubbles by ion adsorption [39]. The nanobubbles displaying a higher absolute zeta potential value showed a good stability and constant nanobubble diameter for a long period. On the other hand, with a low zeta potential value, the nanobubble size quickly increased and disappeared. The zeta potentials of 8% H_2_ in Ar, CO_2_, and the mixture of 8% H_2_ in Ar and CO_2_ gas nanobubbles in water as a function of pH are shown in Figure 2 [35], where the isoelectric point (IEP) is in between 5 and 6. As shown in Figure 2, in the ethanol aqueous solution of less than 50 vol%, the zeta potentials of nanobubbles are also close to zero at pH 5. To maintain the stability of nanobubbles, the pH of ethanol aqueous solution containing nanobubbles can be adjusted to assign higher absolute zeta potential values on the bubble surface, for instance, at alkaline pH 9 or acidic pH 3. In this experiment, pH 9 was selected to examine the stability of bubbles and fits with the water quality standard pH (from 6.5 to 9.5 in EU directive [40]). The nanobubble stability was also evaluated by extended DLVO theory calculation, which will be discussed in the following Section 3.3.

When CO_2_ nanobubbles are added in 8% H_2_ in Ar nanobubbles containing aqueous solution, the CO_2_ solubility is high in aqueous solution and HCO_3_^−^ ion is produced according to the acidity constant. The HCO_3_^−^ ions are adsorbed on the positively charged bubbles. While in the alkaline region, CO_3_^2−^ ion is also produced according to equilibrium constant; however, anions are not adsorbed on the negatively charged bubbles. This agreed with the CO_2_ solubility phenomena explained in the literature [35].

**Figure 3:**
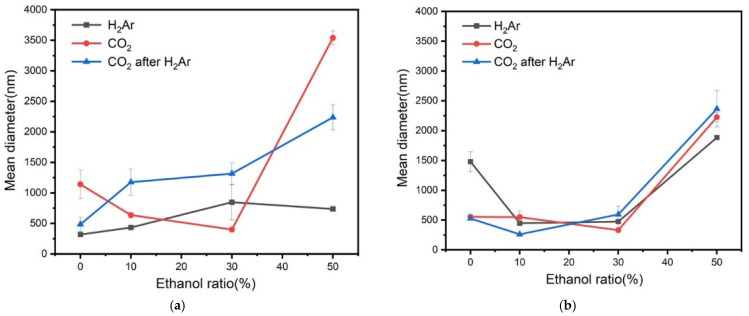
Mean diameter of several nanobubbles in ethanol aqueous solution at pH 9 after (**a**) 1 and (**b**) 20 days.

The nanobubble mean diameters at pH 9 after 1 and 20 days are shown in Figure 3. The diameters of 8% H_2_ in Ar nanobubbles in ethanol aqueous solutions with various ethanol vol.% in the first day by controlling pH 9 are small and do not change significantly (between 300 and 750 nm). On the other hand, CO_2_ and CO_2_ after 8% H_2_ in Ar had noticeably large diameters at 50 vol.% ethanol aqueous solution (2000–3500 nm). The CO_2_ nanobubbles undergo mass loss at a higher pH, corresponding to the mass transfer process owing to the concentration gradient at the surrounding nanobubbles, and their mean diameter decreased [41]. Figure 3 shows that the CO_2_ diameter decreased after 20 days, in 0 vol.% (from 1300 to 600 nm) and 50 vol.% ethanol (from 3500 to 2200 nm). The nanobubble size at the CO_2_ after 8% H_2_ in Ar was larger at 10 and 30 vol% ethanol on the first day (1300 nm) than at 20 days (250–600 nm). It can be explained by Ostwald ripening [42], increasing the nanobubble size, and the size decreased after 20 days. After 20 days, the three kinds of nanobubble mean diameters existed from 300 to 600 nm in 10 and 30 vol% ethanol aqueous solution.

### 3.2. Effect of Ethanol Ratio in Zeta Potential and pH of Nanobubbles

**Figure 4:**
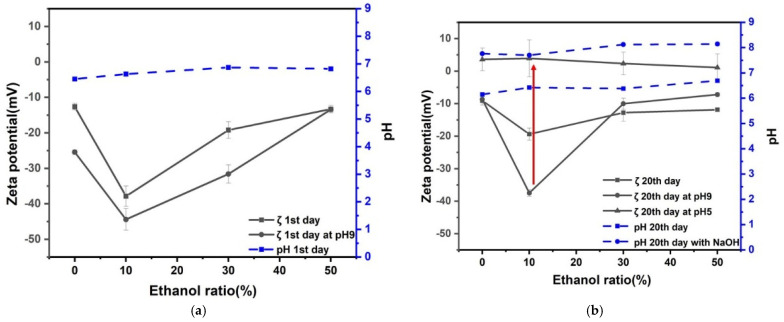
Zeta potential and pH for 8% H_2_ in Ar nanobubbles as a function of ethanol percentage of ethanol aqueous solution mixtures after: (**a**) 1 day and (**b**) 20 days.

Zeta potential and pH for 8% H_2_ in Ar nanobubble solution as a function of ethanol percentage in ethanol aqueous solution mixture after 1 and 20 days are shown in Figure 4. The natural pH of nanobubble solutions was around pH 6 to 7 after 1 and 20 days. Once the solution pH was adjusted to pH 9 by adding NaOH aqueous solution after the first day; it decreased to pH around 8 after 20 days, and the absolute value of the negative zeta potential decreased. The hydrogen solubility in ethanol is explained by Henry’s law [31]. The pH solutions at around pH 8 were adjusted to 5 by adding HCl aqueous solution. The zeta potential of 8% H_2_ in Ar nanobubbles at pH 5 was positive of a few mV, regardless of ethanol percentage. In particular, the zeta potential deviation was the largest (between −45 and 5 mV) at 10 vol% ethanol aqueous solution during the above-mentioned conditioning procedures. Thus, the radical production by 8% H_2_ in Ar nanobubbles in 10 vol% ethanol aqueous solution was investigated by changing the pH from 9 to 5 after 20 days, and the results will be discussed in Section 3.5.

**Figure 5:**
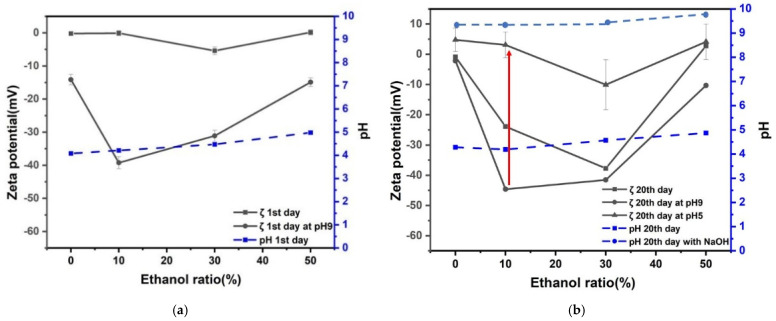
Zeta potential and pH for CO_2_ nanobubble solution as a function of ethanol percentage of ethanol aqueous solution mixtures after: (**a**) 1 day and (**b**) 20 days.

Zeta potential and pH of CO_2_ nanobubble solution as a function of ethanol percentage in ethanol aqueous solution mixtures after 1 and 20 days are shown in Figure 5. The natural pH of nanobubble solutions was around pH 4 to 5 after 1 and 20 days. Once the solution pH was adjusted to pH 9 by adding NaOH aqueous solution on the first day, the solution pH increased to between 9 and 10 after 20 days, and the absolute value of negative zeta potential increased 5 to 10 mV at 10 to 30 vol% ethanol aqueous solution. Our results agreed with the literature. The pH of the aqueous solution containing CO_2_ gas nanobubbles slightly increased after several days compared with the pH under the initial condition [35]. Dalmolin et al. [32] showed that the CO_2_ solubility in ethanol aqueous solution increased by increasing the ethanol mole fraction and pressure and decreasing the temperature.

The pH of solutions with pH around 8 were adjusted to pH 5 by adding HCl aqueous solution. The zeta potential of CO_2_ nanobubbles at pH 5 was a few negative mV. In particular, the zeta potential deviation was the largest (between −45 and 5 mV) at 10 vol% ethanol aqueous solution during the above-mentioned conditioning procedures. This can be explained by the bubble collapse. Therefore, the radical production by CO_2_ nanobubbles in 10 vol% ethanol aqueous solution was investigated by changing the pH from 9 to 5 after 20 days, and the results will be discussed in Section 3.5.

**Figure 6:**
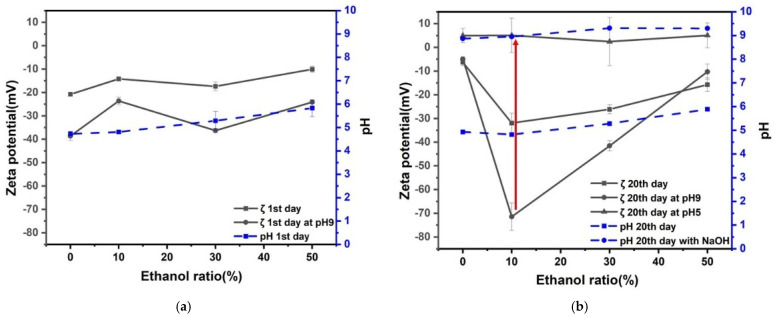
Zeta potential and pH of CO_2_ after 8% H_2_ in Ar nanobubble solution as a function of ethanol percentage of ethanol aqueous solution mixtures after: (**a**) 1 day and (**b**) 20 days.

Zeta potential and pH of the mixture of CO_2_ and 8% H_2_ in Ar nanobubble solution as a function of ethanol percentage in ethanol aqueous solution mixtures after 1 and 20 days are shown in Figure 6. The natural pH of nanobubble solutions was around pH 5 to 6 after 1 and 20 days. On the other hand, once the solution pH was adjusted to pH 9 by adding NaOH aqueous solution on the first day, the solution pH increased to between 9 and 9.5 after 20 days, and the absolute value of negative zeta potential increased from 10 to 30 vol.% ethanol aqueous solution (−40 to −50 mV).

The pH of the solutions at around pH 9 were adjusted to pH 5 by adding HCl aqueous solution. The zeta potential of nanobubbles at pH 5 was slightly positive (i.e., about 5 mV), regardless of ethanol percentage. In particular, the zeta potential deviation was the largest (between −50 and 5 mV) at 10 vol% of ethanol aqueous solution during the above-mentioned conditioning procedures, similar to other results shown in Figure 4 and Figure 5. The radical production by CO_2_ and 8% H_2_ in Ar nanobubbles in ethanol aqueous solution was investigated by changing the pH from 9 to 5 after 20 days, and the results will be discussed in Section 3.5.

The three kinds of well stabilized nanobubbles (8%H_2_ in Ar, CO_2_, and CO_2_ after 8%H_2_ in Ar) at pH 9 in the 10 vol.% ethanol solution displayed decreases in the zeta potential to near zero when adjusting pH to 5, and during such pH adjustment, there was a possibility to produce the radicals originated from the nanobubble collapse. This point will be further discussed in the following Section 3.3 (nanobubble stability), Section 3.4 (nanobubble number), and Section 3.5 (radical production).

### 3.3. Nanobubble Stability Evaluation Using Extended DLVO Theory

Among many kinds of stabilization models for nanobubbles, Tan et al., 2021 suggested that the charge stabilization model can provide reasonable and consistent explanations [15]. 

In this study, bubble stabilization was evaluated by the extended DLVO theory using our experimental results of bubble size (Figure 3) and zeta potential (Figure 4, Figure 5 and Figure 6). Two bubble interactions can be expressed by extended DLVO theory [35,43,44]. Here, the extended DLVO theory is utilized as qualitative analysis. When two same radii (*a*) of nanobubbles are set at the surface-to-surface distance (*h*) between them in the ethanol aqueous solution, the total potential energy (*V_T_*) can be the sum of van der Waals interaction energy (*V_A_*), hydrophobic interaction energy (*V_H_*), and the electrostatic interaction energy (*V_R_*). *V_T_* is described in Equations (1) and (2), normalized by the absolute temperature (*T*) and Boltzmann constant (*k*_B_).

(1) $$\frac{V_{\;T}}{k_{B}T}=\frac{V_{A}+V_{H}+V_{R}}{k_{B}T}$$

(2) $$V_{A}+V_{H}=-\frac{A+K}{6}\left[\frac{2a^{2}}{h\left(4a+h\right)}+\frac{2a^{2}}{{\left(2a+h\right)}^{2}}+ln\left\{\frac{h\left(4a+h\right)}{{\left(2a+h\right)}^{2}}\right\}\right]$$

where the Hamaker constant is *A* and hydrophobic constant is *K*.

The Hamaker constant *A* for air in water (air-water-air value) is 3.7 × 10^−20^ J [45]. As the Hamaker constant *A* is proportional to the surface tension of solvent, 2.2 × 10^−20^, 1.7 × 10^−20^, and 1.4 × 10^−20^ J were used as *A* for air in 10, 30, and 50 vol.% ethanol aqueous solution, respectively. Hydrophobic constant *K* was estimated at 10^−17^ J in the absence of salt and 10^−19^ J in a 1 mM NaCl aqueous solution [44]. In a 1 M ethanol aqueous solution, *K* is about 3 to 7 × 10^−17^ J, which is 3 to 7 times larger than *K* in water [46]. In this article, at 10 vol.% ethanol aqueous solution at pH 9 and 0.01 mM, 1 × 10^−17^ J was used as *K*.

When the surface charge of nanobubbles is Ψ, κ  is the Debye–Hückel parameter; $\epsilon_{r}\;\mathrm{and}\;\epsilon_{o}$ are relative permeability and space permeability, respectively; and $V_{R}$ is shown in Equations (3) and (4).

(3) $$\;V_{R}=\pi \epsilon_{r}\epsilon_{o}a\psi^{2}\left[ln\frac{1+\exp\left(-\kappa h\right)}{1-\exp\left(-\kappa h\right)}+ln\left\{1-\exp\left(-2\kappa h\right)\right\}\right]$$

(4) $$\kappa =\sqrt{\frac{2\pi z^{2}e^{2}}{\varepsilon_{r}\epsilon_{o}kT}}$$

where *n* is the concentration of anions or cations in the solution and is equal to 1000 *N*_A_*C* (*N*_A_ is the Avogadro’s number and *C* is concentration in mol/L), *z* is the valence of ion, *e* is the electron charge, and the thickness of the electric double layer of the nanobubble is Debye length = 1/κ.

**Figure 7:**
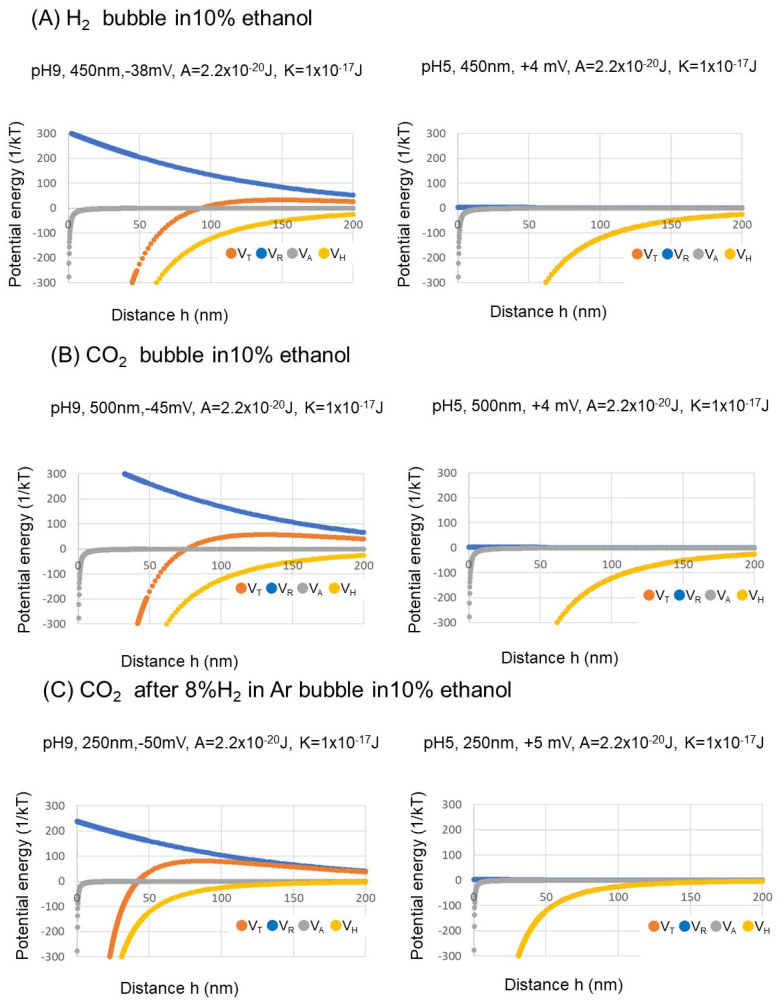
Total potential energy (*V_T_*), electrostatic interaction energy (*V_R_*), van der Waals interaction energy (*V_A_*), and hydrophobic interaction energy (*V_H_*) as a function of the surface-to-surface distance between two nanobubbles at pH 9 and pH 5 of three kinds of gas nanobubbles in 10 vol.% ethanol aqueous solution.

The total potential energy based on electrostatic interaction energy, van der Waals interaction energy, and hydrophobic interaction energy as a function of the bubble distance at pH 9 and pH 5 of three kinds of gas nanobubbles (i.e., 8%H_2_ in Ar, CO_2_, and CO_2_ after 8%H_2_ in Ar)) is shown in Figure 7. The total potential energy barriers at pH 9 of three types of nanobubbles exist with a high negative zeta potential. The various potential energies in the −38, −45, and −50 mV zeta potential of 8% H_2_ in Ar, CO_2_, and CO_2_ after 8% H_2_ in Ar nanobubbles at pH 9 are shown (A, B, C) in Figure 7, respectively. The extended DLVO theory was utilized as qualitative explanation between the bubble’s stability in this paper. The retardation in van der Waals potential can be estimated at least at a longer separation distance than about 15 nm according to Israelachvili, 1985 [47]. Between hydrophobic surfaces, very-long-range attraction can be observed in Figure 7, as also reported for separation [48].

The long-distance hydrophobic forces are evident. In contrast, the total potential is larger than 20 k_B_T at more than 100 nm distance and maintains the stability of bubbles owing to the strong repulsion explained by high electrostatic interaction energy. The maximum total potential energy *V_T_* 15 k_B_T would be the boundary to determine coagulation or dispersion [49]. The total potential energy barrier appeared at 8% H_2_ in Ar, CO_2_, and CO_2_ and 8% H_2_ in Ar nanobubbles at 10 vol.% ethanol aqueous solution at pH 9 (Figure 7). On the other hand, at pH 5, the absolute zeta potential value becomes less than 5 mV for three kinds of gas nanobubbles and total potential energy barrier disappeared owing to negligible electrostatic repulsion (*V_R_*, Figure 7). Therefore, at pH 5, nanobubbles would break owing to the low bubble surface charge, and thus make larger bubbles by bubble coalescence. Zhang et al., 2011 suggested that a higher than 20 vol.% ethanol solution may remove the nanobubbles and cause them to disappear, and is related to the long-range hydrophobic force with ethanol contents [18]. At 50 vol.% ethanol aqueous solution, the zeta potentials for three kinds of nanobubbles initially controlled at pH 9 were about −10 mV after 20 days, and bubble size was large—around 2000 nm—as shown in Figure 3, and not stable.

### 3.4. Number of Nanobubbles

**Figure 8:**
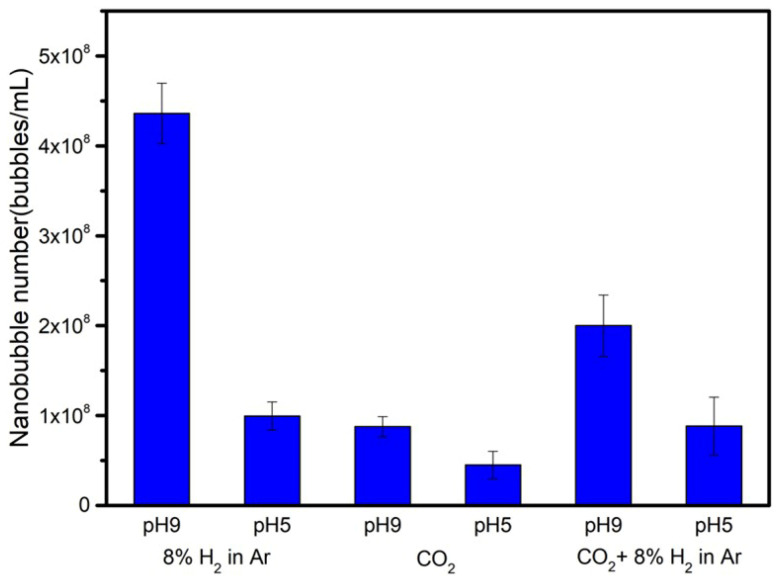
Nanobubble numbers at pH 9 and pH 5 for 8% H_2_ in Ar, CO_2_, and CO_2_ after 8% H_2_ in Ar in 10 vol.% ethanol aqueous solution mixture after 20 days.

The nanobubble numbers for three kinds of gas (8% H_2_ in Ar, CO_2_, and CO_2_ after 8% H_2_ in Ar) in 10 vol.% ethanol aqueous solution mixtures were investigated at pH 9 and pH 5 by nano site after 20 days, and the results are shown in Figure 8. The nanobubble number decreases from pH 9 to pH 5 for each nanobubble solution. In particular, the nanobubble number of 8%H_2_ in Ar decreased the largest from 4 × 10^8^ to 1 × 10^8^ bubbles/mL. As the nanobubble number decreased from pH 9 to pH 5, the nanobubbles became larger by their coalescence and were broken. When the nanobubbles were broken, there was a possibility to produce radicals. Takahashi et al., 2021, reported that ∙OH was generated from the collapsing microbubbles, including oxygen with 2 vol% ozone in 1 mM FeSO_4_ aqueous solution under strongly acidic conditions without ultrasound or a large pressure difference [25]. The following Section 3.5 will report and discuss the experimental results on radical observation as a function of solution pH.

### 3.5. Radical Observation by Changing the pH

**Figure 9:**
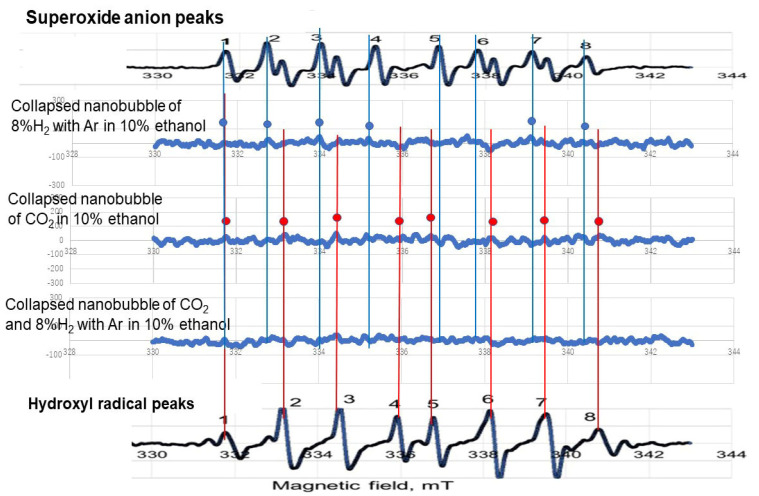
Radical observation by changing pH from 9 to 5 of three kinds of gas nanobubbles (i.e., 8% H_2_ in Ar, CO_2_, and CO_2_ after 8% H_2_ in Ar) in 10 vol.% ethanol aqueous solution mixture.

Figure 9 shows the two standard data points (i.e., superoxide anion on the top, hydroxyl radical on the bottom) and three data points from our gas bubble solutions (i.e., 8% H_2_ in Ar, CO_2_, and CO_2_ after 8% H_2_ in Ar). The ·O_2_^−^ and ·OH and standard eight peaks by spin trap reagent G-CYPMPO appear under different magnetic fields [38]. They are plotted in the top and bottom of Figure 9, respectively. The peaks appearing of 8% H_2_ with Ar, CO_2_, and the mixture of CO_2_ and 8% H_2_ with Ar nanobubbles in 10% ethanol aqueous solutions are plotted as the second, third, and fourth curves from the top in Figure 9, respectively. The peaks corresponding ·O_2_^−^ and ·OH^−^ are marked by blue and red circles, respectively. In this experiment, small peaks were observed in each position owing to smaller nanobubble numbers after 20 days passed.

Radical observation by changing the solution pH from 9 to 5 of three kinds of gas nanobubbles in 10 vol.% ethanol aqueous solution mixture is shown in Figure 9. The pH of the 8% H_2_ in Ar nanobubbles by changing to 5 (second data point from the top) showed a small amount of superoxide anion peaks. The reaction is considered as Equation (5):

(5) 2H_2_O + H_2_ → ·O_2_^−^ + 3H_2_

The CO_2_ nanobubbles, by changing to pH 5 (third data point from the top), showed a small amount of hydroxyl radical peaks. The reaction is shown as follows:

(6) 2OH^−^ + CO_2_ → ·OH + HCO_3_^−^

On the other hand, the mixture of CO_2_ and 8% H_2_ in Ar (fourth data point from the top) showed neither ·O_2_ nor ·OH peaks.

(7) 2H_2_O + H_2_ → ·O_2_^−^ + 3H_2_ → 2·OH + 2H_2_

(8) 2·OH + 2H_2_ + 2CO_2_ → 2·CO_3_H + 2H_2_ → 2H_2_CO_3_ +H_2_

When the acid was added to the nanobubble solution, 8% H_2_ in Ar nanobubble solution produces ·O_2_^−^, while the CO_2_ nanobubble solution produced ·OH. On the other hand, the gas mixture (8% H_2_ in Ar and CO_2_) nanobubbles were prepared by 8% H_2_ in Ar gas blowing followed by CO_2_ gas blowing. At first, the reaction in Equation (7) occurred, and then the reaction in Equation (8) occurred and reduced the radicals. This reaction agreed with the literature. The existing ·OH and ·O_2_^−^ scavenging was reported by blowing CO_2_ nanobubbles after blowing H_2_ nanobubbles [30].

**Figure 10:**
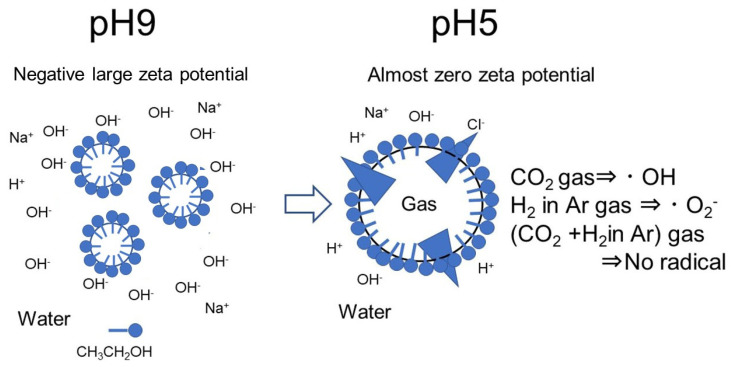
Model of nanobubble breakage by changing pH from 9 to 5 and the production of radicals by decreasing the bubble zeta potential absolute value.

The formation of a monolayer by ethanol [12] and the arrangement of ethanol molecules on interfaces stabilize the bulk nanobubbles [13,14]. The water molecules were arranged to maximize the hydrogen bonding between the oriented ethanol and the adjacent water molecules [16]. Our model of nanobubble increases with coagulation by changing pH from 9 to 5; therefore, the bubble becomes easily breakable, and the production of radicals is shown in Figure 10. Here, the weak ultrasonic wave and ultraviolet light are irradiated. There is a report that the ∙OH peak was observed with a weak supersonic wave [28]. Moreover, ∙OH was generated from the collapsing microbubbles under strongly acidic conditions without any dynamic stimulus such as ultrasound [25]. The CO_2_ nanobubbles after 8% H_2_ in Ar nanobubbles can exist in 10 vol.% ethanol aqueous solution mixture at pH 9 for a long period such as 20 days (Figure 3). The alkaline 10 vol.% ethanol aqueous solution with CO_2_ nanobubbles after 8% H_2_ in Ar nanobubble did not show noticeable free radicals by changing the pH to acidic (i.e., pH 5). The phenomena studied and discussed in this article would be useful to prepare a soda alcohol beverage, among others.

## 4. Conclusions

The 8% hydrogen (H_2_) in argon (Ar) and carbon dioxide (CO_2_) gas nanobubbles with ethanol were produced at 10, 30, and 50 vol.% ethanol aqueous solution by the high speed agitation method with gas injection, and the main findings were as follows:

- The prepared nanobubbles were stable for 20 days owing to a high negative zeta potential at alkaline pH 9.
- When the pH of ethanol alkaline aqueous solution with nanobubbles was adjusted to acidic at around pH 5, the zeta potential of nanobubbles was almost zero. The numbers of nanobubble decreased at almost zero charge (pH 5) were identified by measuring their numbers using the particle trajectory method (Nano site).
- The extended Derjaguin, Landau, Verwey, and Overbeek (DLVO) theory was used to evaluate the nanobubble stability (repulsion between bubbles) in alkaline conditions, and its instability (attraction between bubbles) in acidic conditions.

The collapsed nanobubbles at zero charge generated slight free radicals detected using G-CYPMPO spin trap reagent in electron spin resonance (ESR) spectroscopy. The produced free radicals were superoxide anions at collapsed 8% H_2_ in Ar nanobubbles and hydroxyl radicals at collapsed CO_2_ nanobubbles. On the other hand, the collapsed mixed CO_2_ and H_2_ in Ar nanobubbles showed no free radicals. 

Based on this study, a schematic model of nanobubble breakage and the production of radicals by changing solution pH was proposed. These phenomena and their understanding would be useful to formulate healthy beverages, for example.

## Data Availability

The data presented in this study are available on request from the corresponding author.
